# Skin substitutes as treatment for chronic wounds: current and future directions

**DOI:** 10.3389/fmed.2023.1154567

**Published:** 2023-08-29

**Authors:** Nicole M. Vecin, Robert S. Kirsner

**Affiliations:** ^1^Departments of Medical Education and Public Health Sciences, University of Miami Leonard M. Miller School of Medicine, Miami, FL, United States; ^2^Dr. Philip Frost Department of Dermatology and Cutaneous Surgery, University of Miami Leonard M. Miller School of Medicine, Miami, FL, United States

**Keywords:** skin substitutes, skin grafts, chronic wounds, diabetic foot ulcers, venous leg ulcers

## Abstract

Chronic wounds such as diabetic foot ulcers and venous leg ulcers place a significant burden on the healthcare system and in some cases, have 5-year mortality rates comparable to cancer. They negatively impact patients’ quality of life due to pain, odor, decreased mobility, and social isolation. Skin substitutes are an advanced therapy recommended for wounds that fail to show decrease in size with standard care. The choice of substitute used should be based on evidence, which often differs based on wound etiology. There are more than 75 skin substitutes currently available, and that number is rising. In this review, we discuss current management and future directions of chronic wounds while providing a review of available randomized control trial data for various skin substitutes.

## 1. Introduction

**Figure 1:**
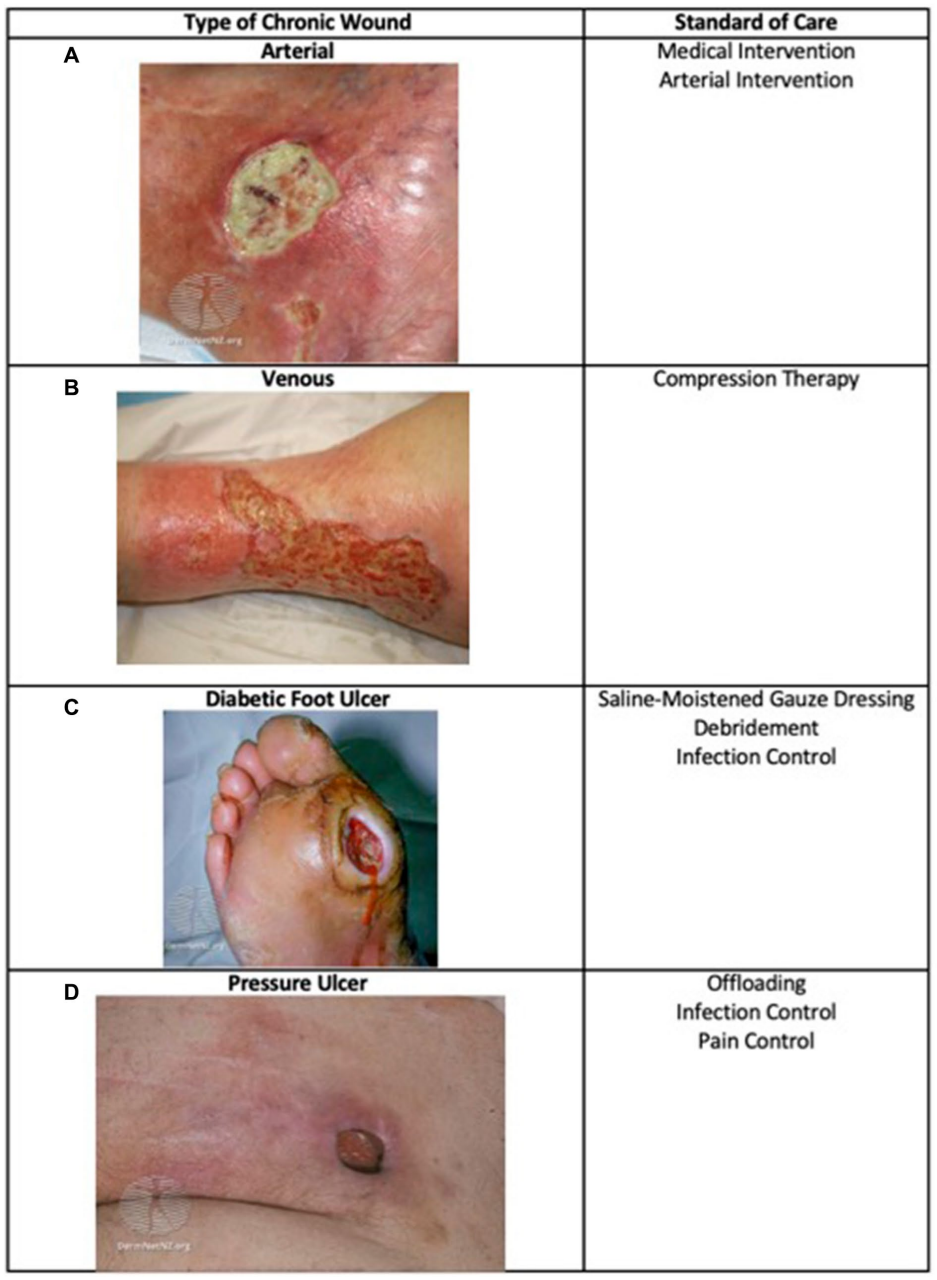
Types of chronic wounds and their standard care. Major types of chronic wounds include arterial, venous, diabetic, and pressure ulcers. The standard care depends on wound etiology (7–10). **(A)** Arterial image: (17) from DermNet (https://dermnetnz.org/topics/martorell-ulcer), licensed under CC BY-NC-ND 3.0 NZ; **(B)** Venous image: (18) from DermNet (https://dermnetnz.org/topics/stasis-ulcer), licensed under CC BY-NC-ND 3.0 NZ; **(C)** Diabetic Foot Ulcer image: (19) from DermNet (https://dermnetnz.org/topics/diabetic-foot-ulcer), licensed under CC BY-NC-ND 3.0 NZ; **(D)** Pressure Ulcer image: (10) from DermNet (https://dermnetnz.org/topics/pressure-ulcer), licensed under CC BY-NC-ND 3.0 NZ.

A wound is a disruption of normal anatomic structure and function (1). Acute wounds typically resolve in 4–6 weeks while chronic wounds persist after initial injury (2, 3). Chronic wounds affect 2% of the U.S. population, approximately 8.2 million Medicare beneficiaries, and are a significant burden on the healthcare system, costing an estimated $28 billion each year (4). The most common types of chronic wounds are due to vascular disease (such as venous or arterial ulcers), due to changes in the nervous system (such as diabetic neuropathic ulcers and pressure ulcers), or a combination (such as diabetic neuroischemic ulcers) (5, 6) (Figure 1). Complications of chronic wounds include osteomyelitis, amputation, and sepsis and some ulcers such as diabetic foot ulcers (DFUs) increase mortality as they have 5-year mortality rates comparable to some cancers (3, 11). Chronic wounds negatively impact patients’ quality of life due to pain, odor, reduced mobility, and social isolation (12). Depression and anxiety often accompany chronic wounds and may subsequently further impact endocrine and immune function (13).

Advanced therapies such as the use of skin substitutes are recommended for wounds that fail to decrease in size 4 weeks after injury (14). Skin substitutes are a diverse group of products that serve as a temporary or permanent coverage of a wound and promote wound healing through various mechanisms (15). Skin substitutes may work in part by promoting wound healing by protecting the integument from loss of fluids, preventing infection, providing a stable, often biodegradable, scaffold that promotes synthesis of new dermal tissue, allowing host cells to proliferate within the scaffold as functional dermal cells rather than scar tissue, delivering or augmenting production of cytokines and growth factors, and must resist shearing force (16).

Replacing skin is not new as the first reports of skin grafting date back to 2,500 B.C. India where Susruta, the Father of Surgery, created the “Ancient Indian Method” that was employed to treat ulcers on the extremities as well as facial deformities (20). Autologous skin grafts were used primarily until the 19th century when xenografts were developed, followed by the first skin allograft transplantation in the 1870s by Thiersch and a report on the utility of skin grafts in healing by Reverdin (21–23). Around this time, the concept of epithelial cell seeding was coined by Mangoldt, but it was not until 1975 that epithelial cell culture was successfully conducted by Rheinwald and Green. The first artificial dermal substitute was developed in the 1980s by Burke et al. for use in burn patients and it became known as the Integra Dermal Regeneration Template. Following this milestone, composite grafts were introduced in the 1990s and the term “tissue engineering” was coined (24, 25).

**Figure 2:**
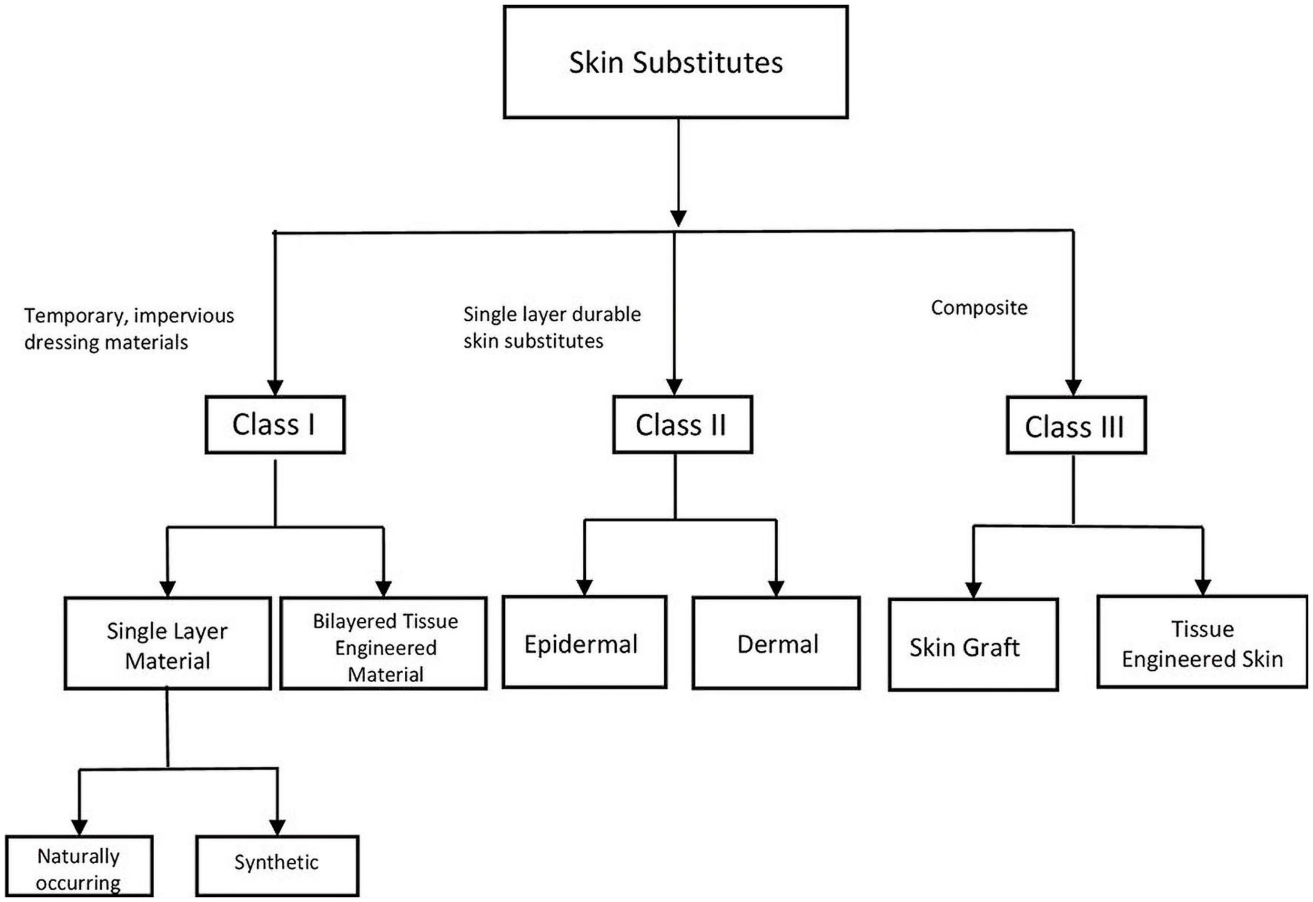
Visual representation of Kumar’s classification system. Kumar’s classification system is a widely used, imperfect classification system for organizing skin substitutes. The classification system denotes three classes. Class I is comprised of temporary, impervious dressing materials and is further divided into single layer and bilayer substitutes. Single layer substitutes may be naturally occurring or synthetic. Class II substitutes are single layer, durable skin substitutes which are either dermal or epidermal. Class III substitutes are composite substitutes that contain both epidermal and dermal layers.

Since then the field has exploded and according to data collected by the federal Agency for Healthcare Research and Quality (AHRQ), the number of commercially available skin substitutes is 76, and that number continues to rise (23). This number of available products is daunting, and several classification systems have been developed to categorize products and thus make it easier for clinicians to determine which substitute is fitting for their patient. Skin substitutes may be classified based on their cellularity (cellular vs. acellular), the layer(s) of skin the substitute is designed to replace (dermal, epidermal, or both), or whether they are derived from natural or synthetic sources. Due to the complexity and redundancy of categorization systems, it remains challenging to organize skin substitutes into their respective categories. An imperfect but widely used skin substitute classification system was created by Kumar in 2008 and divides skin substitutes into three classes (Figure 2) (24). Class I is comprised of temporary, impervious dressing materials and is further divided into single layer and bilayer substitutes. Single layer substitutes may be naturally occurring or synthetic. Class II substitutes are single layer, durable skin substitutes which are either dermal or epidermal. Class III substitutes are composite substitutes that contain both epidermal and dermal layers (24).

The U.S. Food and Drug Administration (FDA) is responsible for overseeing the introduction of new drugs and devices to the U.S. market through the evaluation of a product’s safety and efficacy, and assessment of risks and benefits regarding their use (25). Skin substitutes are typically regulated under one of the following regulatory pathways: Human Cells, Tissues, And Cellular and Tissue-Based Products (HCT/Ps), Premarket Approval (PMA), 510(k) Premarket Notification, or Humanitarian Device Exemption (HDE) (26, 27). The regulatory pathway in which a substitute follows is determined based on the substitute’s class. Medical devices fall under 3 classes. Categorizing devices into their respective class depends on their potential risk and intended use (28). Highest risk medical devices, such as implantable devices, are categorized under Class III devices whereas devices that are low risk are Class I (29). Skin substitutes may be categorized as either Class I, II, or III depending on their composition and use. Most Class I and many Class II devices are exempt from further scrutiny. Devices that are not exempt require a 510(k) Premarket Notification (28). Class III devices are those that may support or sustain human life, prevent impairment of health, or potentially pose a significant risk to human health and require PMA (30). PMA involves stringent evaluation by the FDA to determine there is adequate scientific evidence to ensure a product is both safe and effective (31). The Bilayered Cellular Construct (BLCC, Apligraf®, Organogenesis, Canton, MA) is an example of a skin substitute with PMA (32). 510(k) Premarket Notification is a submission made to the FDA before the product goes on the market in which the company compares the safety and efficacy to an equivalent product that is currently on the market and in which the safety data is already known, known as a predicate device. The new device will gain approval if it is deemed “Substantially Equivalent (SE)” to a predicate device (33). An HDE is rare, and it is intended for devices that are used for the treatment or diagnosis of conditions that do not affect more than 8,000 individuals in the U.S. each year (27). These devices are exempt from stringent evaluation due to their potential impact in the management of a rare disease. Skin substitutes containing human cells or tissue, such as Porcine Small Intestine Submucosa (SIS, OASIS®, Smith and Nephew, Ft Worth, TX) are regulated as HCT/Ps under the Title 21of Code of Federal Regulations, part 1271 (21 CFR 1271) to confirm safety and good tissue practices (34). Placental derived and amniotic fluid based products are regulated under either section 361 or section 351 of the Public Health Service Act. Section 361 allows use of amniotic products for homologous use only, meaning they can be used only for repair, reconstruction, replacement, or supplementation of a recipient’s cells or tissues when applied using implantation, transplantation, infusion, or transfer into a human recipient.

The current review will discuss current management and future directions of DFUs and venous leg ulcers (VLUs) and will focus on skin substitutes with randomized controlled trial (RCT) data. Given the lack of data for pressure ulcers we will not discuss these.

## 2. Classification

### 2.1. Class I

Class I skin substitutes are comprised of single and bilayer temporary, impervious dressing material. They aim to maintain mechanical traits of the skin but are acellular. They may be naturally occurring or synthetic. Naturally occurring skin substitutes include human amniotic membranes. Class I skin substitutes are advantageous in protecting against infection and water loss. They help maintain a moist environment that is conducive to wound healing (35).

There are several amnion-derived products that are used in the treatment of chronic wounds. Placental membranes have been historically recognized for their regenerative potential, with the first transplanted amniotic membrane occurring in 1910 (36). The placenta consists of an inner amnion on the fetal side of the placenta and the outer chorion on the maternal side of the placenta. The amnion in contact with the fetus contains epithelial cells that could serve as a source of stem cells. Beneath this is a basement membrane consisting of collagen, laminins, and fibronectin, fibroblasts within matrix and a basement membrane for the underlying chorion. The amnion contains a number of growth factors and cytokines that are crucial in wound healing. Amnion-derived substitutes convey the advantage of pain relieving qualities, antibacterial and non-immunogenic properties, reduction in inflammation and scar development, and provide a matrix for migration and proliferation of cells (37). Disadvantages of amnion-derived substitutes include costs and fragility (38). Synthetic Class I substitutes will not be discussed due to their lack of evidence.

### 2.2. Class II

Class II dressings are single layer durable skin substitutes. They can be further categorized as epidermal or dermal. Examples of epidermal substitutes include epidermal sheets (EpiDex®, Anika Therapeutics, Inc., Bedford, MA, United States and BioSeed-S®, BioTissue Technologies AG, Freiberg, Germany) while dermal substitutes include SIS and Dermal Skin Substitutes (DSS, Dermagraft®, Organogenesis, Canton, MA, United States). As examples these single layer substitutes were designed to replace a single layer of skin.

#### 2.2.1. Epidermal

Epidermal substitutes are structured to resemble epidermis and serve to replace solely the epidermis. Cultured epithelial autografts (CEAs) are epithelial skin substitutes that are derived from autologous keratinocytes that are grown *ex vivo* in culture with murine fibroblasts. The patient’s keratinocytes are grown and replicate to form a dressing several layers thick (39).

The keratinocytes cultured to construct epidermal substitutes may arise from various sources, including the patient’s skin or hair follicles (39, 40). EpiDex® is an epidermal equivalent derived from the isolated keratinocytes of the outer root sheath of anagen hair follicles (40). These isolated keratinocytes are then cultured onto a sheet that is applied to the wound (41). Laserskin is an epidermal substitute that is derived from autologous keratinocytes that are cultured onto a hyaluronic acid membrane containing perforations to allow proliferation of cells (41). BioSeed-S® is an epidermal substitute derived from autologous keratinocytes and fibrin sealant (41).

Epidermal substitutes have shown some promise in the treatment of VLUs and DFUs however the challenges associated with their use make them an unpopular choice in the treatment of chronic wounds. A major limitation of epidermal substitutes is that they lack the mechanical stability and elasticity that the dermis provides. This makes them fragile, unreliable, and challenging to care for (38, 42). Drawbacks to the use of these substitutes is that they are prone to breakdown, blistering, and result in scar formation (24). In addition, the time required to create CEAs provides a challenge for those patients requiring an immediate intervention, as they take approximately 3–4 weeks to construct (43). For these reasons, the epidermal substitutes EpiDex®, Laserskin, and BioSeed-S® are not commercially available at this time, though many may be available through direct contact with their manufacturer.

#### 2.2.2. Dermal

Dermal substitutes provide several advantages over epidermal substitutes such as their ease of use and durability. They may also produce better quality scarring and minimize the risk of contracture (24). In addition, acellular dermal material may be used which provides the advantage of being non-immunogenic (24). Acellular dermal substitutes may be composed of naturally occurring polymers such as collagen, elastin or hyaluronic acid, synthetic polymers, porcine dermis, or de-epithelialized cadaveric skin, as examples (44). A major disadvantage of dermal substitutes includes their cost (38). Graftjacket® (Wright Medical Technology, Memphis, TN, United States) and DermACELL® (LifeNet Health, Virginia Beach, VA, USA) are both human acellular dermal matrices (ADM) and as such, are regulated under 21 CFR Part 1271 Part 361 Human Cells, Tissues, and Cellular and Tissue-based Products (HCT/Ps) (45).

MatriStem MicroMatrix® (ACell Inc., Columbia, MD, United States) is an ADM derived from porcine urinary bladder matrix (UBM). This UBM has U.S. FDA 510(k) Premarket Notification and is indicated for partial and full-thickness wounds, pressure ulcers, VLUs, DFUs, chronic vascular ulcers, tunneled undermined wounds, surgical wounds, traumatic wounds, and draining wounds (46, 47). AlloPatch® (Musculoskeletal Transplant Foundation Sports Medicine, Edison, NJ, United States) is an open-structure human reticular acellular dermal matrix (HR-ADM) that has been evaluated for its use in DFU treatment (48, 49). This HR-ADM is regulated under 21 CFR Part 1271 Part 361 Human Cells, Tissues, and Cellular and Tissue-based Products (HCT/Ps) (45). Hyalograft 3D (Anika Therapeutics, Inc., Bedford, MA, United States) is an autologous fibroblast-hyaluronic acid complex dermal substitute (50).

DSS is a cryopreserved human fibroblast-derived dermal substitute that is derived through the culture of neonatal dermal fibroblasts onto a bioabsorbable mesh scaffold (51). Fibroblasts incorporated onto the scaffold promote wound healing by secreting various growth factors, cytokines, proteins, and collagen. DSS currently has U.S. FDA PMA and is indicated for full thickness DFUs with greater than 6 weeks duration that extend through the dermis but without tendon, muscle, joint capsule, or bone exposure. DSS substitutes should be used in conjunction with SOC and in patients with adequate blood supply (52, 53).

SIS is an acellular biological extracellular matrix that contains several factors that promote wound healing such as collagen, elastin, glycosaminoglycans, proteoglycans, and growth factors (54). Growth factors mitigate the destruction by matrix metalloproteinases (MMPs) and also induce angiogenesis. One consideration and potential drawback in the use of porcine SIS substitutes includes their cultural acceptability. Facilitation of open discussion with the patient can assist in navigating these challenges and identifying alternative treatments when appropriate (55). SIS is regulated under 21 CFR Part 1271 Part 361 Human Cells, Tissues, and Cellular and Tissue-based Products (HCT/Ps) (45). Wound Conforming Matrices (WCM) composed of type I bovine fibrillar collagen provide another dermal substitute alternative (56).

## 3. Class III

Class III skin substitutes consist of composite substitutes that contain both epidermis and dermis. They can be further divided into autografts, allografts, xenografts, and tissue engineered skin. Cadaveric skin allografts and xenografts have been predominantly used in the management of burn wounds while tissue engineered skin substitutes such as BLCC, Dermal Regenerative Template (DRT, Integra® Integra LifeSciences, Plainsboro, NJ, United States), and Biobrane (UDL Laboratories, Inc., Rockford, IL, United States) have shown efficacy in chronic wounds.

### 3.1. Autografts

Autografts are derived from a patient’s healthy skin. They may be used for either acute or chronic wounds such as burns and traumatic wounds or large or refractory chronic wounds (57). Challenges in autograft use include the introduction of a new wound, contracture and scar formation, infection and bleeding risk, and decreased or increased sensation, as well as the necessity and cost of an operating room and hospitalization depending on graft size (58). Despite these challenges, autografts provide the advantages of availability and decreased immunogenicity (59). Autografts may be full-thickness, containing epidermis and full dermis, or split-thickness, containing epidermis and superficial dermis (57). While autologous grafts take, allogeneic grafts are rejected. Graft rejection is a term used to describe the rejection of transplanted host cells by donor immune cells due to genetic discrepancies between the host and donor (60). However, autografts may fail to “take,” or incorporate into the recipient site. Take occurs in three consecutive phases including plasmatic imbibition, inosculation, and revascularization (61). Autografts, as opposed to flaps, do not contain their own blood supply and are initially ischemic and pale in color. In order to receive nutrients, plasmatic imbibition occurs in which oxygen and nutrients are absorbed from the underlying wound bed (62). Inosculation describes the following phase in which capillaries from the graft establish connections with the underlying wound bed to provide a blood supply to the graft. At this time, the autograft may appear pink. Lastly, revascularization of the graft occurs. Failure to progress through these phases may result in graft failure that presents clinically as pale white in color or as black eschar 1–2 weeks after placement (63).

As the main challenge of autografts involves donor site morbidity and disfigurement, one method has been developed to mitigate this. Micro skin tissue columns (MTSCs) are full-thickness microscopic skin transplants harvested from healthy skin that are less than 1 mm in diameter (59, 64, 65). Standard hypodermic needles are used to extract the skin column from the donor site. A fluidic device is then used to remove the column from the needle (65). MTSCs are spread evenly on the wound bed. Even when several MTSCs are harvested from the donor site, due to their small size disfigurement is minimal to nonexistent and donor site morbidity is greatly decreased. It is known that chronic wounds remain in dysregulated inflammation in which a resolved inflammatory phase is not achieved due to either excessive or suboptimal inflammation (66). Recent studies give evidence that promoting progression through the inflammatory phase of wound healing in these stalled wounds provides healing benefit (67–69). MTSCs promote wound healing through acceleration of reepithelialization and contraction, epidermal differentiation, increased dermal collagen, and attenuation of the inflammatory response in order to resolve inflammation (59). MTSCs provide an innovative and promising approach to autologous skin grafts.

### 3.2. Allografts

Cadaveric skin allografts are derived from donated skin that is preserved via cryopreservation or glycerol preservation and stored in a tissue bank. Cadaveric skin allografts provide patients with viable epidermis and dermis. Cadaveric skin allografts have been used as temporary biologic dressings in extensive burn injuries in order to prepare the wound bed for a subsequent permanent split-thickness autologous graft and have also been explored for their use in the treatment of chronic wounds. The allograft functions to form a matrix for the formation of granulation tissue (70). Benefits of cadaveric skin allografts include decreased pain, stimulation of angiogenesis, prepares the wound bed for autografting, infection control, low cost, and availability (70). Theraskin (LifeNet Health, Virginia Beach, VA, United States) is an example of a cadaveric cryopreserved human skin allograft (CHSA) product and is regulated under 21 CFR Part 1271 Part 361 Human Cells, Tissues, and Cellular and Tissue-based Products (HCT/Ps) (45). Challenges in the use of cadaver skin allografts is potential transmission of various diseases such as cytomegalovirus, which has been seen in cadaveric skin allograft transplantation in burn wounds, as well as longevity of the grafted skin (71, 72).

Human skin allografts may also be donated from a living donor, though they are not commonly used in the treatment of chronic wounds. An important consideration in the use of human skin allografts, whether preserved cadaveric or derived from a living donor, is immunogenicity. When treating a wound with living donor skin, close relation between the donor and recipient confers decreased risk of immunogenicity and prolonged graft survival (73). Though cadaveric skin consists of dead cells, some cells may retain major histocompatibility class II (MHC Class II) molecules which enables rejection and thus processing is critical to prevent stimulation of the immune system (74). Immunogenicity occurs is thought to occur through the action of immune cells such as dendritic cells, Langerhans cells, T cells, B cells and NK cells. Skin contains resident immune cells such as these in all layers. Notably, the epidermis plays a critical role in immunity by housing CD8+ T cells, Langerhans cells, and dendritic cells and secreting antimicrobial peptides (AMPs), defensins, and damage-associated molecular patterns (DAMPs) as well as pro-inflammatory cytokines such as IL-1 and TNF-α (75). In allogeneic skin graft transplantation, donor dendritic and Langerhans cells from the grafted skin migrate to recipient lymph nodes and present donor antigens to recipient T cells, known as direct allorecognition (76, 77). Recipient T cells and B cells may also be activated from donor antigens on recipient antigen presenting cells, known as indirect allorecognition (78, 79). NK cells are thought to also play a role in rejection through the killing of donor cells through antibody-dependent cellular toxicity (76, 80). Similar to solid organ transplantation, strategies are employed to prevent skin allograft rejection. Systemic immunosuppression use confers increased risk such as systemic or wound infection (81). If immune suppression is not used, pretreatment of the allograft to reduce the function of immune cells such as Langerhans and dendritic cells provides a method to reduce immune reactivity in skin transplantation (81, 82). Chemical agents or short wavelength UV irradiation may be used to deplete dendritic cells or inhibit antigen presentation (83–86). Additionally, oxidative damage may be prevented through pretreatment with antioxidants (87). Other studies provide evidence for the bioengineering modification of grafts to evade the recipient immune system (88). With these innovative approaches, these temporary dressings may be beneficial in promoting wound healing (89).

### 3.3. Xenografts

Skin xenografts, similar to cadaveric allografts, may be used as a temporary, initial treatment that prepares the wound bed for autologous transplantation. When using xenografts, factors such as immunogenicity and disease transmission must be considered. In order to mitigate disease transmission, rigorous processing must typically occur (90). This processing has the capacity to damage and denature the xenograft structure. However, xenografts confer the advantages of being lower cost, in unlimited supply, and having lower risk of disease transmission than their allograft counterparts (89). Xenografts used in the treatment of chronic wounds include porcine, bovine, and more recently, fish (91, 92). A major advantage of the use of fish skin grafts over other types of xenografts include lower risk of viral transmission, enabling mild processing that preserves skin integrity which contains beneficial collagen, elastin, and Omega-3 polyunsaturated fatty acids thought to improve reepithelialization and microbial defense (90, 93, 94).

### 3.4. Tissue engineered skin

In the early 1990s, the term “tissue engineered skin” was coined to describe the creation of a product comprised of human skin and bio-scaffolds that have the capacity to replace damaged human skin while resembling its natural function and structural characteristics, such as maintaining flexibility, acting as a protective barrier, and preventing transepidermal water loss (95). Examples of composite tissue engineered skin include BLCC and DRT. The major limitation of this subtype of composite substitutes is cost, as these products are expensive to produce (96). DRT is a bilayer regeneration matrix comprised of a dermal layer and an overlying silicone layer. The dermal layer is comprised of an acellular matrix consisting of cross-linked bovine collagen and chondroitin-6-sulfate, a type of glycosaminoglycan. A thin silicon layer overlays the dermal layer and acts as the epidermis (97).

BLCC is a widely used composite tissue engineered skin substitute. It is a bilayer living cellular construct that consists of two components. The dermal component is comprised of bovine type I collagen and human neonatal foreskin fibroblasts while the epidermal component is comprised of keratinocytes (98). BLCC has U.S. FDA PMA and is indicated for use with standard DFU care for the treatment of full-thickness neuropathic DFUs of greater than 3 weeks duration which have not adequately responded to standard therapy and extend through the dermis but without tendon, muscle, joint capsule, or bone exposure. In addition, it is indicated for use with standard therapeutic compression for the treatment of non-infected partial and thickness skin ulcers due to venous insufficiency of greater than 1 month duration which have not adequately responded to conventional ulcer therapy (32, 99). The mechanism of action of BLCC in VLUs has been studied on a molecular level. RCTs comparing BLCC to compression therapy alone found that BLCC decreases expression of profibrotic TGF-β1 and increased levels of TGF-β inhibitor and also upregulated matrix metalloproteinases to stimulate antifibrotic remodeling (68). In addition, a RCT comparing healing of venous ulcers treated with BLCC vs. compression therapy alone enabled transcriptomic analysis of wounds of each treatment modality. BLCC treated wounds had three distinct transcriptomic patterns that suggest a shift from a non-healing to healing tissue response, resembling that of an acute healing wound (67).

## 4. Evidence for skin substitutes

In the current section, the authors will present RCT data available for the various substitutes in order to assist clinicians in determining which substitute is appropriate for their patient. As the quality of trials differ, we provide the sample size (n) as one rough surrogate for quality. RCT data are summarized in Table 1.

**Table 1:** Evidence for available skin substitutes used to treat chronic wounds.

	Class	Type of wound	Number of participants (*n*)	Comparison	Healed (%)	Time to closure (days)	FDA status	Effective-ness data
dHAM (100–102)	I	DFU	29	SOC*	33 vs. 0 at 6 weeks		HCT/P	+
		DFU	13	SOC*		29.5		
vCPM (103–106)	I	VLU	21	SOC*	53 vs. 0 at 10.9 weeks		HCT/P	+
		DFU	26	SOC*	65.4 vs. 0 at 12 weeks	34		
		DFU	97	SOC*	62 vs. 21 at 12 weeks	42		
		DFU	62	DSS	48.4 vs. 38.7 at 8 weeks			
dHACM (102, 107–111)	I	DFU	60	BLCC	85 vs. 35 (BLCC) vs. 30 (SOC*) at 4 weeks	13	HCT/P	+
				SOC*				
		DFU	100	BLCC	97 vs. 73 (BLCC) vs. 51 (SOC*)	23.6		
				SOC*				
		DFU	110	SOC*	70 vs. 50 at 12 weeks			
		VLU	109	SOC*	60 vs. 35 at 12 weeks			
DermACELL® ADM (112)	II	DFU	168	GraftJacket® SOC*	65 vs. 41.1 (SOC*) at 12 weeks (No difference compared to Graft-jacket)	63	HCT/P	+
GraftJacket® ADM (112)	II	DFU	168	DermACELL® SOC*	No difference compared to DermA-CELL® or SOC*		HCT/P	+
SIS (54, 55, 113–117)	II	VLU	120	SOC*	55 vs. 34 at 12 weeks		HCT/P	+
		Mixed VLU and arterial	54	Hyaloskin®	82.6 vs. 46.2 at 16 weeks			
		Mixed VLU and arterial	50	Petrolatum-Moistened Gauze	80 vs. 65 in 8 weeks			
		DFU	55	DSS	73.7 vs. 47.1 (DSS) vs. 57.9 (SOC*) at 12 weeks			
				SOC*				
UBM (118)	II	DFU	56	DSS	No significant difference		510(k)	+
HR-ADM (49)	II	DFU	40	SOC*	65 vs. 5 at 6 weeks	28	HCT/P	+
DSS (119–122)	II	DFU	46	SOC*	71.4 vs. 14.3 at week 12		PMA	+
		VLU	18	SOC*	50 vs. 12.5 at 12 weeks			
		VLU	366	SOC*	34 vs. 31 at week 12			
CHSA (123)	III	DFU	23	DSS	63.6 vs. 33.3 at 12 weeks	62.3	HCT/P	+
BLCC (124–130)	III	DFU	72	SOC*	51.5 vs. 26.3 at 12 weeks	84	PMA	+
		DFU	208	SOC*	56 vs. 38 at 12 weeks	65		
		DFU	17	SOC*	56 vs. 37 at 12 weeks			
		Arterial	31	SOC*	62 vs. 0 and 8 weeks	49		
		VLU	120	SOC*	47 vs. 19 in 6 months	181		
DRT (112–114, 131–133)	III	DFU	307	SOC*	51 vs. 32 at 16 weeks	43	PMA	+

The table depicts RCT data for available skin substitutes that are currently used in the treatment of chronic wounds. *SOC is used to denote standard of care. DFU, diabetic foot ulcer; VLU, venous leg ulcer; dHAM, dehydrated human amniotic membrane; vCPM, cryopreserved placental membrane containing viable cells; dHACM, dehydrated amnion and chorion membrane; ADM, acellular dermal matrices; SIS, small intestine submucosa; UBM, urinary bladder matrix; HR-ADM, human reticular acellular dermal matrix; DSS, dermal skin substitutes; CHSA, cryopreserved human skin allograft; BLCC, bilayered cellular construct; DRT, dermal regenerative template; SOC, standard of care; HCT/P, human cells, tissues, and cellular and tissue-based products; 510(k), 510(k) premarket notification; PMA, premarket approval.

### 4.1. Class I: amnion-derived

RCTs have evaluated the efficacy of amnion-derived substitutes as treatment for DFUs. These trials indicated that amniotic substitutes serve as effective treatment for DFUs. Serena et al. (*n* = 76) found that a hypothermically stored amniotic membrane increased frequency and probability of wound closure in DFUs as compared to the standard of care (134). A RCT by Snyder et al. (*n* = 29) found that a dehydrated human amniotic membrane (dHAM), known as AmnioExcel® Amniotic Allograft Membrane (Integra LifeSciences, Plainsboro, NJ, United States), along with the standard of care led to more robust healing than the standard care alone (100). When evaluated with concurrent use of a total contact cast (*n* = 13), patients treated with dHAM and total contact cast had higher healing rates and lower recurrence rates than those who were treated with a total contact cast and standard care (101). Two trials evaluated the use of a cryopreserved placental membrane containing viable cells (vCPM, Grafix®, Osiris Therapeutics, Inc., Columbia, MD, United States) for treatment of DFUs (*n* = 26, *n* = 97) and one trial evaluated its use in the treatment of VLUs (*n* = 21). These trials deemed vCPM more effective in treating their respective ulcers than the standard of care (103–105). A 2018 RCT (*n* = 62) compared the efficacy of vCPM and DSS, a dermal cellular construct with proven efficacy and effectiveness for DFUs, in the treatment of DFUs. The Osiris sponsored trial’s preliminary results indicated that vCPM may have better outcomes for wounds 5 cm or smaller and allows significant cost savings compared to DSS (102, 106). vCPM, like many placental products, is regulated under 21 CFR Part 1271 Part 361 Human Cells, Tissues, and Cellular and Tissue-based Products (HCT/Ps) (45).

EPIFIX® (MiMedx Group, Marietta, GA, United States) is a matrix comprised of dehydrated amnion and chorion membrane (dHACM). It is also regulated under 21 CFR Part 1271 Part 361 Human Cells, Tissues, and Cellular and Tissue-based Products (HCT/Ps) (45). RCTs have evaluated its effectiveness in treating DFUs. In this MiMedix sponsored trial (*n* = 60, *n* = 100), dHACM proved to be superior to BLCC with efficacy and effectiveness data for DFUs and VLUs (Organogenesis, Inc., Canton, MA) in treating DFUs as the dHACM treated group had a higher proportion of complete wound closure and had significantly faster wound healing rates. In addition, the number of grafts and graft cost per patient were considerably lower than the BLCC group (107, 108). In further evaluation of their efficacy in the treatment of DFUs, Tettelbach et al. (*n* = 110) conducted a RCT comparing dHACM to the standard of care and results indicated that dHACM along with the standard of care improved healing (109). RCT data (*n* = 40) supports the use of dehydrated human amniotic chorionic membrane (dHACM) as weekly treatment for DFUs as opposed to biweekly for optimal results (110). dHACM was also evaluated for the treatment of VLUs. dHACM in conjunction with compression therapy was compared to the standard care of compression alone (*n* = 109). Results indicated that dHACM is advantageous as an adjunctive therapy for non-healing venous leg ulcers (111).

### 4.2. Class II: epidermal

The following epidermal substitutes are not currently available but are mentioned here in order to present their efficacy and due to their potential availability through direct contact with the manufacturer.

A RCT (*n* = 77) conducted in 2003 compared epidermal sheets to the mesh graft in treatment of chronic leg ulcers. Results showed that at week 12, complete closure rates were similar between the two arms however, in those patients that did not have complete closure at week 12, the epidermal sheet group had greater reduction of wound area and these patients underwent continuous healing (40).

A RCT (*n* = 225) analyzed time to healing and number of healed ulcers in two groups of patients with non-healing venous ulcers. One group was treated with epidermal sheets in conjunction with compression while the other group was treated with the standard of care which consisted of compression alone. Epidermal sheets depicted an advantage over the standard of care in healing of venous leg ulcers with a statistically significant difference in time to healing (135).

Clinical trials have shown the efficacy and safety of CEAs in the treatment of DFUs and VLUs. A 2012 RCT randomized diabetic foot ulcer patients (*n* = 63) into two treatment arms, one received a CEA substitute (*n* = 31) while the other received gauze with Vaseline (*n* = 32). Complete healing was achieved in 100% of the treatment group and 69% of the control group (50). CEAs showed faster healing of venous leg ulcers when paired with compression than with compression alone (136, 137).

### 4.3. Class II: dermal

A RCT (*n* = 168) evaluating the wound healing potential of acellular dermal matrices randomized patients into three treatment arms, DermACELL® ADM (LifeNet Health, Virginia Beach, VA, United States), GraftJacket® ADM (Wright Medical Technology, Memphis, TN, United States), or standard therapy. GraftJacket® ADM is an acellular dermal matrix similar to DermACELL® but is freeze-dried. Therefore, it requires time for rehydration prior to use. Wounds treated with a single application of DermACELL® ADM showed greater wound closure rates than the standard care. GraftJacket® ADM did not show greater wound closure rates than the standard care at any time point. Compared to standard care and GraftJacket® ADM, DermACELL® ADM depicted greater healing, wound area reduction, and a longer duration of closure (112).

In 2019, a RCT (*n* = 55) showed that SIS and DSS, both dermal substitutes, had comparable healing rates with no observable differences in wound closure and percentage of wound area reduction in diabetic foot ulcers (113). SIS has been shown to improve healing of VLUs in conjunction to compression therapy when compared to compression therapy alone (*n* = 120) (114). It proved superior to Hyaloskin, an extracellular matrix containing only hyaluronic acid, in treatment of hard-to-heal arterial and venous ulcers in parameters such as healing time, time to dressing change, pain, and comfort (*n* = 54) (115). It also proved superior to moist wound dressings in the healing of arterial ulcers and VLUs (*n* = 50) (116). As far as cost effectiveness, a RCT found that SIS as an adjunctive treatment to the standard of care for DFUs improves outcomes and is more cost-effective than the standard of care alone (117).

In a 2016 RCT (*n* = 95), the wound healing capability of UBM was compared to DSS dermal substitute in the treatment of DFUs. Outcomes analyzed included incidence of ulcer closure, rate of ulcer healing, wound characteristics, quality of life, cost effectiveness, and recurrence. An interim analysis showed that UBM provided similar healing outcomes to DSS at a lower cost and increased quality of life (118).

In a RCT (*n* = 40), HR-ADM was evaluated for its efficacy in facilitating closure of DFUs as compared to the collagen-alginate standard dressing. At 6 weeks, 65% of DFUs treated with HR-ADM healed as compared to 5% treated with standard care with no difference in adverse events thus proving that weekly application of HR-ADM is an effective intervention for treatment of DFUs (49).

RCTs have shown superiority of DSS in conjunction with standard of care as compared to the standard of care alone in the treatment of DFUs (119, 120). The use of DSS as adjunctive treatment to the standard of care was shown to have a decreased incidence of amputation and bone resection (*n* = 314) (119). An early 2002 trial (*n* = 46) established its effectiveness and safety in treating DFUs (120). DSS does not appear efficacious in the treatment of venous leg ulcers as the results of a 2004 RCT (*n* = 18) indicated that DSS was shown to promote increased healing in conjunction with compression when compared to compression alone but a larger 2013 RCT (*n* = 366) found no significant difference in healing rates between the control and treatment groups (121, 122).

The utility of hyaluronic based autologous fibroblast dermis (AFD, Hyalograft 3D, Anika Therapeutics, Inc., Bedford, MA, United States) in treating DFUs was explored in a 2014 clinical trial (*n* = 63) in which the efficacy and safety of AFD was compared to a non-adherent foam dressing. Complete ulcer healing was achieved in 84% of patients treated with AFD and 34% of patients treated with a non-adherent foam dressing. In addition, healing times were faster for AFD as compared to the non-adherent foam dressing (36.4 ± 17.6 vs. 48.4 ± 13.1 days). There was no difference in incidence in adverse events per group (50). Another RCT (*n* = 180) compared AFD with the standard of care for dorsal or plantar DFUs. Patients were randomized to receive either AFD first followed by epidermal sheets or AFD followed by a non-adherent paraffin gauze. Study results demonstrated that at 12 weeks, complete healing was similar in both groups but a 50% reduction in ulcer area was achieved significantly faster in the treatment group. This provides evidence for the use of AFD followed by epidermal sheets (138). These results point toward AFD as an effective intervention for treatment of DFUs.

A RCT (*n* = 41) was conducted to evaluate the effectiveness of a topically applied GAM501 (Ad5PDGF-B/Bovine Type I Collagen Gel) WCM in treating DFUs as compared to the standard of care. Patients underwent a 2 week run-in period where they received the standard of care which included daily saline-moistened gauze dressing changes. Patients with wounds that did not reduce in area by more than 30% were randomized to continue receiving the standard of care or to receive the WCM. The results of this RCT demonstrated that WCM promoted significant acceleration of wound healing as compared to the standard of care and was not associated with adverse events. The WCM provides a treatment intervention for DFUs (56).

### 4.4. Class III: allograft

In 2014, Sanders et al. conducted a RCT (*n* = 23) to assess healing outcomes of DFUs treated with CHSA vs. DFUs treated with DSS dermal substitutes. Results of this RCT supported CHSA as superior to DSS in healing and suggest that DFUs managed with CHSA were twice as likely to heal than those treated with DSS (123).

### 4.5. Class III: xenograft

Xenografts are most commonly porcine but recent studies showed utility in the use of fish skin xenografts as compared to the standard of care for the treatment of DFUs and VLUs (93). Results showed that wounds treated with fish skin grafts healed over 50% sooner than the control group and in less than 10% of the time than was predicted. Of those wounds treated with fish skin grafts that did not achieve the required skin reduction in weeks 4–8, these wounds still ultimately healed. In this study (*n* = 42), fish skin grafts are the recommended treatment as per Swiss guidelines for wounds that do not improve by 40%–50% in 4 weeks of standard care. In addition, a 2020 study (*n* = 170) created wounds in healthy volunteers that served to imitate chronic wounds and compared healing outcomes between Atlantic cod fish skin grafts and dHACM. Results supported the superiority of fish skin grafts over dHACM as wounds treated with fish skin grafts healed significantly faster (90).

### 4.6. Class III: tissue engineered skin

A multitude of RCTs have been conducted to assess the efficacy, safety, and effectiveness in treating chronic wounds such as VLUs and DFUs. When compared to the standard of care, DFUs treated with BLCC showed greater rates of wound closure, with comparable safety metrics to the standard (124–127). A RCT (*n* = 31) evaluating BLCC as treatment for ischemic wounds compared to the standard moistened dressings found that treatment with BLCC promotes healing more rapidly and in more patients (128). Falanga et al. conducted trials (*n* = 120, *n* = 240) to assess the use of BLCC as treatment in VLUs as compared to standard care. Results indicated that BLCC is highly effective in treating VLUs, especially those of long duration (129, 130).

In 2015, a RCT (*n* = 307) was conducted to assess the safety and efficacy of DRT in the treatment of DFUs as compared to a control group that was treated with 0.9% sodium chloride gel, a secondary dressing, and offloading. Results of this trial showed that complete diabetic foot ulcer closure was significantly greater in the treatment group, the rate of wound size reduction per week was greater than the control, patient quality of life was improved, and DRT proved to have less severe adverse events as compared to the control (131). A 2021 RCT (*n* = 36) evaluated the efficacy of DRT alone vs. DRT with negative pressure therapy (NPT) in healing wounds with exposed bone or tendon. Outcomes assessed included time to take of the graft and time to skin transplantation. Results indicated that using NPT as an adjunctive therapy to DRT increased the take rate and had the capacity to decrease hospital stays (132). DRT has U.S. FDA PMA and is indicated for post-excisional treatment of life-threatening full-thickness or deep partial thermal injuries where sufficient autograft is not available at the time of excision or not desirable due to the physiological condition of the patient and repair of scar contractures (46, 133).

## 5. Future directions

### 5.1. 3D printing

Each skin substitute discussed above has its respective limitations, whether it be risk of disease transmission, immunogenicity, financial cost, or cultural acceptability. For these reasons, there is need for new skin substitutes that mitigate these challenges. In recent years, the emergence of 3D printing in advanced tissue engineering has provided a potential solution to fill the need for a cost effective, efficacious treatment modality (139, 140). A major shortcoming of current skin substitutes is that they are not capable of replacing skin appendages such as hair follicles, vasculature, and glands and often heal with inadequate pigmentation. 3D printing has the potential to produce these appendages and create a realistic, functional substitute (139, 141). The development of 3D printed substitutes may eliminate the need for autologous grafting and may even surpass current healing techniques (140, 142).

### 5.2. Vascularization

Despite current advances made in tissue engineering, one area in need of innovation is the vascularization of skin substitutes. Vascularization is essential for the diffusion of nutrients, oxygen, and immune reactors into the area of interest. Large defects may provide challenges regarding nutrient and oxygen diffusion and as a result, nutrient deprived cells that are distant from surrounding capillaries experience impaired proliferation and migration (143). One solution to augment vascularization is the prevascularization approach in which skin substitutes are vascularized prior to application on the wound bed (22, 144). This may be achieved through the use of growth factors or stem cells that promote angiogenesis in the skin substitute or through techniques that prevascularize skin substitutes, such as 3D printing (143, 145). This method has shown efficacy in a murine model (146). A bilayer printed graft containing printed endothelial cells showed a 10% increase in wound contraction as compared to controls an on histology, the printed graft closely resembled normal skin (146). Consideration of vascularity in the development of skin substitutes provides a promising new approach in tissue engineering.

### 5.3. Stem cells

Another potential direction to pursue in tissue engineering includes the implementation of stem cells in skin substitutes due to their potential for accelerated wound closure, reduced scar formation, and regeneration of skin appendages that are lacking in current skin substitutes (147). Stem cells have been shown to promote the secretion of cytokines and growth factors that stimulate angiogenesis and extracellular matrix (ECM) remodeling in the wound bed (148). Several types of stem cells, including bone marrow-derived mesenchymal stem cells, bone marrow-derived endothelial progenitor cells, hematopoietic stem cells, and adipose-derived stromal cells, have been studied for their potential use in wound healing (149–152). A recent study has compared the use of human neonatal stem cells (hNSCs), amniotic epithelial stem cells (AECs), and mesenchymal stem cells (MSCs) derived from placenta and results show promise in wound healing. hNSCs, AECs, and MSCs were isolated from placenta, differentiated into keratinocytes and fibroblasts, and mixed in plasma to create a skin substitute which was transplanted onto a severe rat thermal wound. The stem cells successfully promoted wound healing through reepithelialization and improved skin architecture (153). A 2019 study investigated the effectiveness of various types of stem cells in regenerating the epidermis of a wound. Adipose-tissue-derived stem cells (ADSCs), dental pulp stem cells (DPSCs), Wharton’s jelly stem cells (WJSCs), and bone marrow stem cells (BMSCs) were placed on top of a dermal substitute and parameters such as epidermal differentiation, matrix synthesis, and immunogenicity through HLA molecule expression were assessed. Of the various types of stem cells, WJSCs showed greatest potential due to their epidermal differentiation and low immunogenicity (154). While the use of stem cells provides an exciting potential new modality, the use of stem cells presents challenges such as identifying the optimal source of stem cells, refining processing and administration, elucidating the reprogramming process of stem cells, as well as future implications of stem cell derived skin such as immunogenicity and tumorigenicity (155). Further research is crucial in establishing stem cell-based skin substitutes as a viable treatment for chronic wounds.

## 6. Discussion

Chronic wounds are those that do not heal in a timely manner and may have various etiologies such as vascular disease, changes in the nervous system, or a combination of etiologies (2, 3, 5, 6). They affect approximately 2% of the population and may provide complications such as osteomyelitis, amputation, and sepsis with some ulcers such as diabetic foot ulcers conferring 5-year mortality rates comparable to cancer (3, 4, 11). Despite their prevalence and severity, they remain difficult to manage. The development of skin substitutes in the 1980s revolutionized the treatment of chronic wounds. Since then, numerous skin substitutes have been implemented, each boasting their respective advantages and disadvantages. The purpose of the present review is to present RCT data to support the use of skin substitutes while also suggesting future directions in tissue engineering. Much of the RCT data available regarding skin substitutes compares a substitute to the standard of care but there is a lack of abundance of literature comparing the efficacy of skin substitutes to one another. Additional RCT data is necessary to compare substitutes and gain understanding of which substitute best suits a specific clinical picture. This data can serve to guide clinicians in their decision-making. In addition, while available skin substitutes show improvement of healing outcomes in several chronic wound types, they provide barriers such as cost and accessibility. The authors of this review introduced several potential future directions in tissue engineering that may serve to create skin substitutes that are effective and mitigate these challenges.

Amnion-derived skin substitutes include dHAM, vCPM, and dHACM and pose the advantages of pain relieving qualities, antibacterial and non-immunogenic properties, and reduction in inflammation and scar development in treatment of DFUs and VLUs but are fragile and costly (100–111). Epidermal substitutes are not commonly used due to their fragility (40, 50, 135–138, 156). Dermal substitutes such as DSS and SIS are commonly used due to their ease of use and reduced scarring and contractures but are costly (49, 54, 55, 112–122). CHSA decreases pain, stimulates angiogenesis, prepares the wound bed for autografting, controls infection, and is low cost but confers the risk of disease transmission (123). Xenografts such as fish skin grafts promote efficient wound healing, but potential of rejection must be considered (90, 93, 94). Lastly, tissue engineered skin substitutes such as BLCC and DRT promote healing but provide the challenge of cost (97, 124, 125, 129, 131, 132, 157).

In addition to current products, there are several prospective future directions for the development of new alternatives. The implementation of stem cells in skin substitutes, prevascularization of substitutes, and 3D printing are methods currently being explored for their wound healing capacity (139–155). These technologies may provide a promising future for wound healing. With a multitude of products on the market, it is challenging to determine which product is appropriate for a given clinical scenario. It is critical that this decision to be evidence-based. This review delineates advantages and disadvantages of several substitutes currently available and provides RCT data to support their efficacy for wound of various etiologies. The authors of this review hope this information can serve to aid clinicians in choosing the substitute that best suits their individual patient.

## Author contributions

NV and RK: integrity and accuracy of this study. NV: study concept and design, drafting of the manuscript, and administrative, technical, or material support. NV and RK: acquisition, analysis, and interpretation of literature, critical revision of the manuscript for important intellectual content. RK: study supervision. All authors contributed to the article and approved the submitted version.

## Conflict of interest

The authors declare that the research was conducted in the absence of any commercial or financial relationships that could be construed as a potential conflict of interest.

## Publisher’s note

All claims expressed in this article are solely those of the authors and do not necessarily represent those of their affiliated organizations, or those of the publisher, the editors and the reviewers. Any product that may be evaluated in this article, or claim that may be made by its manufacturer, is not guaranteed or endorsed by the publisher.
